# Multivariate random regression analysis for body weight and main morphological traits in genetically improved farmed tilapia (*Oreochromis niloticus*)

**DOI:** 10.1186/s12711-017-0357-7

**Published:** 2017-11-02

**Authors:** Jie He, Yunfeng Zhao, Jingli Zhao, Jin Gao, Dandan Han, Pao Xu, Runqing Yang

**Affiliations:** 1 Freshwater Fisheries Research Centre of Chinese Academy of Fishery Sciences, Wuxi, 214081 China; 2 Key Laboratory of Aquatic Genomics, Ministry of Agriculture; Research Centre for Aquatic Biotechnology, Chinese Academy of Fishery Sciences, Beijing, 100141 China; 3 Department of Biological Science and Technology, Heilongjiang Vocational College for Nationalities, Harbin, 150066 China

## Abstract

**Background:**

Because of their high economic importance, growth traits in fish are under continuous improvement. For growth traits that are recorded at multiple time-points in life, the use of univariate and multivariate animal models is limited because of the variable and irregular timing of these measures. Thus, the univariate random regression model (RRM) was introduced for the genetic analysis of dynamic growth traits in fish breeding.

**Methods:**

We used a multivariate random regression model (MRRM) to analyze genetic changes in growth traits recorded at multiple time-point of genetically-improved farmed tilapia. Legendre polynomials of different orders were applied to characterize the influences of fixed and random effects on growth trajectories. The final MRRM was determined by optimizing the univariate RRM for the analyzed traits separately via penalizing adaptively the likelihood statistical criterion, which is superior to both the Akaike information criterion and the Bayesian information criterion.

**Conclusions:**

In the selected MRRM, the additive genetic effects were modeled by Legendre polynomials of three orders for body weight (BWE) and body length (BL) and of two orders for body depth (BD). By using the covariance functions of the MRRM, estimated heritabilities were between 0.086 and 0.628 for BWE, 0.155 and 0.556 for BL, and 0.056 and 0.607 for BD. Only heritabilities for BD measured from 60 to 140 days of age were consistently higher than those estimated by the univariate RRM. All genetic correlations between growth time-points exceeded 0.5 for either single or pairwise time-points. Moreover, correlations between early and late growth time-points were lower. Thus, for phenotypes that are measured repeatedly in aquaculture, an MRRM can enhance the efficiency of the comprehensive selection for BWE and the main morphological traits.

**Electronic supplementary material:**

The online version of this article (10.1186/s12711-017-0357-7) contains supplementary material, which is available to authorized users.

## Background

From an economical point of view, growth and developmental characters are the most important traits in farmed fish species, and persistent efforts are made to genetically improve these traits in fish breeding. Growth and developmental traits, such as body weight (BWE) and morphological traits, are measured at different times. Faster BWE growth shortens time to market and selection on morphological traits allows fish shape and size to be standardized. Growth and developmental traits are dynamic quantitative traits because they vary spatially and temporally [1]. They are also called infinite-dimensional traits, since they are expressed on a continuous time and space scale [2]. In tilapia breeding, growth and developmental traits at a specific age (in day, week, or year), such as the time to market are the main targets of genetic improvement. As such, genetic analysis of the growth and developmental process can increase the efficiency of selection compared to that of traits measured at specific ages [3].

Considerable attention has been paid to the genetic analysis of BWE and morphological traits at a specific age in breeding tilapia. In earlier studies, growth traits at specific ages were considered as separate traits and analyzed by using univariate animal models [4–13]. Subsequently, multivariate animal models were applied to estimate both the heritabilities of growth traits at specific ages and the genetic correlations between traits measured at different specific ages [8, 11, 12, 14–22]. Although multivariate genetic analysis of growth traits has improved the accuracy of parameter estimation by using more records, at multiple ages, such an analysis is strongly limited by the variable and irregular timing of these measurements [23].

For growth and developmental traits that are recorded at multiple time-points during growth, the patterns of growth curves have been shown to be heritable [2, 3, 24]. The genetic analysis of dynamic quantitative traits was initially conducted by first fitting individual growth curves and then analyzing their estimated parameters within the framework of a multivariate animal model. However, with such genetic analyses, it was not possible to determine whether some individuals had too few records to fit the growth curves. Since then, random regression models (RRM) [3, 25] were developed to dissect dynamic phenotypes into different functions that describe the effects of various genetic and environmental factors. Only three papers on the application of RRM for the genetic analysis of growth curves have been published, in rainbow trout [26] and tilapia [9, 27]. The use of RRM allows heritabilities of growth traits at any age to be estimated, as well as genetic correlations between pairwise traits measured at different ages. Moreover, multiple trait RRM (MRRM) can estimate genetic correlations between pairs of traits measured at different ages and improve heritability estimates for each trait. This not only facilitates the genetic analysis of dynamic traits, but also improves the prediction of breeding values [3, 28].

The objective of this study was to construct and implement a MRRM to simultaneously model the genetic changes in growth traits and estimate the heritability of each growth trait, as well as the genetic correlations between pairs of growth traits measured at specific ages, such as the time to market for a population of genetically improved farmed tilapia in order to formulate a selection criterion for simultaneously selecting BWE and morphological traits. For this purpose, BWE, body length (BL), and body depth (BD) were measured six times on 1451 fish from 45 mixed families of full and half-sibs.

## Methods

### Experimental population

The base population of 1800 one-month old tilapia fingerlings (sex ratio of 1:1) was imported from WorldFish, Malaysia, in 2006, and was derived from 60 families with complete pedigree. The fish were systematically selected for seven generations at the experimental station of the Freshwater Fisheries Research Center in Wuxi, China. Based on breeding values for BWE at 120 days of age estimated using an animal model, 40 broodstocks per family were chosen at each generation, with a sex ratio of 1:1. The selection intensity in males and females was ~ 5%. Broodstocks from different families were randomly mated with each other and 100 to 125 families were retained in each generation. There was one generation of selection per year. In the seventh generation, in 2014, 120 males and 120 females from different families (one male and one female from each family) were selected as experimental parents. In May of that year, each male and two females ready to spawn were maintained in a 1-m^3^ fiberglass tank for one week. Next, 120 females with fertilized eggs in their mouths were separately placed into multiple hapas (1 m × 1 m × 1 m) in a large concrete pond (50 m × 7 m × 1 m) for a one-week incubation period and were then isolated from their progenies. At 50 days of age, 50 families, each consisting of no less than 1000 surviving progenies above 30 g, were used to construct the experimental population. At the same time, 32 progenies that were randomly chosen from each family were tagged with passive integrated transponder tags, and 1600 tagged fish were mixed in a larger concrete pond (50 m × 20 m × 1.8 m). Because of this, we did not include any effect of the rearing facilities on the measurements in the statistical model. In the subsequent experiment, all fish were fed on a standard commercial diet (crude protein: 28%, crude lipids: 4%, crude fiber: 15%, ash: 18%, total phosphorus: 1%, lysine: 1.2%) manufactured by the Feedstuff Incorporated company (Ningbo, China). Dissolved oxygen was maintained at 8.23 to 8.67 mg/L by air pumps and the water temperature naturally fluctuated between 24.3 and 26.7 °C during the entire experiment.

The tagged fish were weighed on electronic scales to measure BWE, while BL and BD were measured with calipers. Before each measurement, the fish were anesthetized with clove oil at a density of 100 mg/L. The fish were measured once every 15 days during the experiment, with the first recording at the time of tagging. A maximum of six records for each fish and each trait were available, since 4.6% of the individuals died before the end of the experiment. Although experimental fish were measured only six times, the traits were recorded on 20 different days of age because of different spawning times. A total of 7560 records were extracted for BWE, BL and BD.

To edit the dataset, fish with missing phenotypic records for any trait and no information on the parents were excluded and abnormal phenotypic values that were more than three standard from the phenotypic mean of the trait were removed. After editing, 1451 individuals with 7235 records remained, with each individual having at least two records per trait. Table 1 presents the descriptive statistics for the dataset for BWE, BL, and BD for the 20 age groups.

**Table 1:** Averages and standard deviations (in parentheses) for body weight (g), body length (mm) and body depth (mm) at 20 days of age

Age	Sample size	BWE	BL	BD
52	473	36.3 (8.5)	88.19 (9.68)	31.74 (3.42)
59	354	49.94 (3.67)	99.28 (3.92)	36.62 (2.00)
65	304	62.47 (12.47)	107.13 (11.46)	41.30 (3.90)
68	469	82.99 (11.00)	116.86 (9.79)	43.71 (2.87)
72	320	95.98 (23.40)	125.40 (12.26)	48.25 (3.52)
75	352	106.19 (10.56)	129.58 (5.23)	49.32 (2.68)
81	302	124.03 (31.99)	133.49 (15.32)	53.66 (4.54)
85	470	138.50 (27.12)	139.96 (11.65)	56.56 (3.74)
88	319	153.00 (40.52)	141.67 (15.28)	60.09 (3.87)
92	352	173.97 (19.17)	149.15 (6.48)	62.43 (2.26)
98	303	200.39 (51.45)	159.64 (19.21)	63.70 (4.61)
102	469	221.12 (44.36)	161.13 (13.83)	64.04 (3.73)
105	319	243.64 (63.82)	164.32 (17.93)	66.17 (4.46)
109	354	275.08 (33.08)	172.25 (8.63)	67.19 (3.04)
115	304	313.77 (77.68)	180.04 (23.21)	69.58 (5.59)
121	473	384.56 (65.23)	185.63 (15.63)	70.32 (4.46)
122	320	391.92 (89.49)	187.85 (19.95)	71.87 (5.06)
128	354	427.41 (105.57)	190.47 (26.18)	72.09 (6.45)
134	304	480.17 (50.97)	194.43 (10.70)	73.15 (3.76)
141	320	538.13 (87.26)	197.54 (17.46)	74.97 (5.21)

Pedigree data were collected by tracing back three generations, thus including 1604 individuals from 77 sires and 88 dams. Although maternal effects on growth traits may be large in the early growth period for tilapia, they were not considered in the analyses, because observations from only a single generation and from such a small number of dams without records render the maternal effects unidentifiable.

### Random regression model

In the same setting of cultivation programs, the two sexes and six test days were considered as fixed effects, while family, additive genetic, and permanent environmental effects were considered as random effects. For the growth-related traits that were recorded at multiple time-points, the phenotype of the trait at $t$ days of age was described by the following single-trait animal model:

1 $$y_{ijkl} \left( t \right) = \mu_{j} + b_{k} \left( t \right) + f_{l} \left( t \right) + a_{i} \left( t \right) + pe_{i} \left( t \right) + e_{ijkl} (t),$$

where $y_{ijkl} \left( t \right)$ is an observation at $t$ *d*ays of age, $\mu_{j}$ is the $j$th test day effect, $b_{k} \left( t \right)$ is the $k$th sex effect, $f_{l} \left( t \right)$ is the $l$th family effect, $a_{i} \left( t \right)$ is the additive genetic effect for the $i$th individual, $pe_{i} \left( t \right)$ is the permanent environmental effect for the $i$th individual, and $e_{ijkl} (t)$ is the residual error following a normal distribution with expectation 0 and variance $\sigma_{e}^{2}$.

To analyze the growth curves of phenotypes that are measured repeatedly, Legendre polynomials are generally used to fit the changes in the fixed and random effects in Model (1) and are represented by:

$$L_{m} \left( t \right) = \beta_{0} + \beta_{1} \psi_{1} \left( t \right) + \cdots + \beta_{k} \psi_{k} \left( t \right) + \cdots + \beta_{m} \psi_{m} (t),$$

where $m$ is the order, $\beta_{k}$ is the $k$th regression coefficient, and $\psi_{k} \left( t \right)$ is the $k$th covariate of the Legendre polynomial [2], defined by:

$$\psi_{k} \left( t \right) = \frac{1}{{2^{k} }}\mathop \sum \limits_{i = 0}^{int(k/2)} \frac{{\left( { - 1} \right)^{i} \left( {2k - 2i} \right)!}}{{i!\left( {k - i} \right)!\left( {k - 2i} \right)!}}\tau^{k - 2i} ,$$

where $$\tau = -\,2 \times \frac{{t - { \hbox{min} }(t)}}{{\hbox{max} \left( t \right)\,\,-\,\,{ \hbox{min} }(t)}}$$.

By modeling changes with age in the effects of sex, family, additive genetics, and permanent environment of the Legendre polynomials with orders $m_{1}$, $m_{2}$, $m_{3}$, and $m_{4}$, respectively, Model (1) becomes the following RRM:

2 $$y_{ijkl} \left( t \right) = \mu_{j} + L_{m1}^{b} \left( t \right) + L_{m2}^{f} \left( t \right) + L_{m3}^{a} \left( t \right) + L_{m4}^{pe} \left( t \right) + e_{ijkl} \left( t \right),$$

where $L_{\eta }^{\theta } \left( t \right) = \beta_{0}^{\theta } + \beta_{1}^{\theta } \psi_{1} \left( t \right) + \beta_{2}^{\theta } \psi_{2} \left( t \right) + \cdots + \beta_{\eta }^{\theta } \psi_{\eta } (t)$, with $\theta \in \left[ {b f a pe} \right]$ and $\eta \in [m_{1} m_{2} m_{3} m_{4} ]$.

In matrix notation, Model (2) can be written as:

3 $${\mathbf{y}}_{ijkl} = \mu_{j} + {\mathbf{x}}_{1i} {\mathbf{b}}_{l} + {\mathbf{x}}_{2i} {\mathbf{f}}_{k} + {\mathbf{x}}_{3i} {\mathbf{a}}_{i} + {\mathbf{x}}_{4i} {\mathbf{pe}}_{i} + {\mathbf{e}}_{ijkl} ,$$

where $${\mathbf{x}}_{\eta i} = [1 \psi_{1} \left( {t_{i} } \right) \ldots \psi_{\eta } (t_{i} )]$$, and $${\varvec{\uptheta}} \in \left[ {{\mathbf{b}}\, {\mathbf{f}} \,{\mathbf{a}} \,{\mathbf{pe}}} \right] = [\beta_{0}^{\theta } \beta_{1}^{\theta } \ldots \beta_{\eta }^{\theta } ]^{T}$$.

The model satisfied the following:

$$\begin{aligned} & E\left( {{\mathbf{y}}_{ijk} |\mu_{j} , {\mathbf{b}}_{l} , {\mathbf{f}}_{k} , {\mathbf{a}}_{i} , {\mathbf{pe}}_{i} } \right) = \mu_{j} + {\mathbf{x}}_{ij} {\mathbf{b}}_{l} + {\mathbf{z}}_{ik} {\mathbf{f}}_{k} + {\mathbf{z}}_{i} {\mathbf{a}}_{i} + {\mathbf{z}}_{i} {\mathbf{pe}}_{i} \\ & Cov\left( {{\mathbf{y}}_{ijk} } \right) = {\mathbf{I}}\,{ \otimes }\,{\mathbf{F}} + {\mathbf{A}}\,{ \otimes }\,{\mathbf{G}} + {\mathbf{I}}\,{ \otimes }\,{\mathbf{PE}} + {\mathbf{I}}\,{ \otimes }\,\sigma_{e}^{2} , \\ \end{aligned}$$

where ${\mathbf{A}}$ is the numerator relationship matrix and ${\mathbf{F}}$, ${\mathbf{G}}$, and ${\mathbf{P}}$ are the family, additive genetic, and permanent environmental covariance matrices for the random regression coefficients of the Legendre polynomials, respectively.

To estimate the genetic correlations between growth traits measured at multiple time-points, the RRM for those traits must be solved simultaneously by a multivariate genetic analysis. The covariance matrices in the MRRM were estimated by using restricted maximum likelihood (REML), as implemented in DMU Version 6 [29]. The starting values were set to 0 for each fixed effect, to the identity matrix for each random effect covariance matrix and to 1 for the residual variance. The convergence criterion for REML was set to 10^−6^.

After estimating the covariance matrices, heritabilities for the traits at any age and the genetic correlations between traits measured at different ages were estimated by the covariance functions [30]. The covariances for a trait between the $i$th and $j$th days of age can be calculated as ${\mathbf{z}}_{i} {\mathbf{Fz}}_{j}^{\text{T}}$ for the family effect, ${\mathbf{w}}_{i} {\mathbf{Aw}}_{j}^{\text{T}}$ for the additive genetic effect, and ${\mathbf{s}}_{i} {\mathbf{PEs}}_{j}^{\text{T}}$ for the permanent environmental effect. Therefore, genetic correlations between the $i$th and $j$th days of age for a trait were estimated by $\frac{{{\mathbf{w}}_{i} {\mathbf{Aw}}_{j}^{\text{T}} }}{{\sqrt {({\mathbf{w}}_{i} {\mathbf{Aw}}_{i}^{\text{T}} )({\mathbf{w}}_{j} {\mathbf{Aw}}_{j}^{\text{T}} )} }}$, phenotypic correlations by $\frac{{{\mathbf{P}}_{ij} }}{{\sqrt {{\mathbf{P}}_{ii} {\mathbf{P}}_{jj} } }}$ with phenotypic covariance ${\mathbf{P}}_{ij} = {\mathbf{z}}_{i} {\mathbf{Fz}}_{j}^{\text{T}} + {\mathbf{w}}_{i} {\mathbf{Aw}}_{j}^{\text{T}} + {\mathbf{s}}_{i} {\mathbf{PEs}}_{j}^{\text{T}} + \sigma_{e}^{2} {\mathbf{I}}(i = j)$, and heritability at the $i$th day of age by $\frac{{{\mathbf{w}}_{i} {\mathbf{Aw}}_{i}^{\text{T}} }}{{{\mathbf{P}}_{ii} }}$.

### Choice of model

When choosing a model for a longitudinal analysis, Stoica and Babu [31] observed that neither the Akaike information criterion nor the Bayesian information criterion had optimum properties in terms of consistency and efficiency, and therefore the authors introduced a new criterion, i.e. penalizing adaptively the likelihood (PAL) [31], that overcomes these issues. More recently, Corrales et al. [32] applied PAL to the selection of the order of the Legendre polynomial in RRM. Consider a set of $m$ competing models $M_{1} , L, M_{k - 1} , M_{k} , L, M_{m}$ that are ranked in ascending order by the number of parameters. The PAL criterion for selecting Model $k$ is defined as:

4 $$PAL_{k} = -\,2\log \left( {ML_{k} } \right) + p_{k} { \log }(p_{m} )\frac{{{ \log }(r_{k} + 1}}{{{ \log }(\rho_{k} + 1)}},$$

where $\log \left( {ML_{k} } \right)$ is the logarithm of the maximum likelihood value of Model $k$; $p_{k}$ is the number of parameters of Model $k$; $p_{m}$ is the largest number of parameters of competing models; and $r_{k} = 2\log \left( {ML_{k - 1} } \right) - 2\log \left( {ML_{1} } \right)$ and $\rho_{k} = 2\log \left( {ML_{m} } \right) - 2{ \log }(ML_{k - 1} )$, with $\log \left( {ML_{1} } \right)$, $\log \left( {ML_{k - 1} } \right)$ and $\log \left( {ML_{m} } \right)$ being the logarithms of the maximum likelihood values of Models $1$, $k {-} 1$, and $m$, respectively.

## Results

### Choice of MRRM

The best MRRM for BWE, BL, and BD was selected by separately optimizing the univariate RRM for growth traits. The Legendre polynomial for the effect of sex was generally modeled by the population’s growth curve, chosen as the Legendre polynomial of three orders according to the highest goodness-of-fit for the three analyzed traits. The three random effects, i.e. family, additive genetic, and permanent environmental effects, was characterized by using Legendre polynomials of different orders that ranged from 0 to 3 [3]. The models were designated as ${\text{LPm}}_{1} {\text{m}}_{2} {\text{m}}_{3}$, e.g., ${\text{LP}}121$ is a model with a Legendre polynomial of order 1 for the family effect, order 2 for the additive genetic effect, and order 1 for the permanent environmental effect. A total of 64 RRM for each analyzed trait were compared based on the PAL criterion to determine the best combination of orders of the Legendre polynomials for the three random effects.

When fitting RRM with constant permanent environmental effects, the variance estimates of these effects were statistically inferred as not significant, using student *t* statistics (estimate/standard error). In addition, RRM with dynamic permanent environmental effects converged poorly in DMU. Therefore, permanent environmental effects were not considered in competing models. In the remainder, for the nine competing models, model LP00, which had constant family and additive effects, was taken as the reference, because the $- 2\log \left( {ML} \right)$ for the RRM with dynamic additive genetic and family effects was consistently lower than for model LP00. Based on Eq. (4), Table 2 presents the calculated PAL values for the nine competing RRM. Among these, ${\text{LP}}23$ was chosen for BWE with Legendre polynomials of order 2 for the family effect and order 3 for the additive genetic effect, while ${\text{LP}}13$ and ${\text{LP}}22$ were selected for BL and BD, respectively.

**Table 2:** Choice of *RRM* for BWE, BL and BD based on the PAL criterion

Model	Parameters	BWE		BL		BD	
		− 2log(ML)	PAL	− 2log(ML)	PAL	− 2log(ML)	PAL
LP00	3	59,206.2		36,641.6		28,386.1	
LP11	11	47,172.4	47,172.4	30,293.0	30,293.0	23,486.4	23,486.4
LP12	16	45,006.1	45,035.6	29,667.0	29,700.4	22,765.9	22,797.9
LP13	22	44,259.8	44,309.2	*29,583.0*	*29,650.3*	22,726.0	22,798.5
LP21	16	46,821.6	46,884.8	30,162.3	30,260.6	23,263.7	23,334.8
LP22	22	44,991.3	45,032.6	29,657.8	29,705.3	*22,745.5*	*22,792.1*
LP23	29	*44,228.7*	*44,294.0*	29,580.0	29,670.8	22,706.3	22,812.4
LP31	22	46,752.8	46,877.5	30,149.3	30,311.3	23,256.0	24,142.4
LP32	29	44,827.3	44,882.0	29,642.1	29,704.9	22,736.9	22,798.5
LP33	37	44,215.4	44,301.9	29,574.4	29,695.9	22,705.9	22,850.5

The italic values represent results of selected RRMs

### Genetic parameters for growth traits

Based on the model choices as described above, the final MRRM was constructed to jointly analyze BWE, BL, and BD. Based on Model (2), the MRRM, in which Legendre polynomials of different orders were nested, was expressed as:

5 $$\left\{ {\begin{array}{*{20}c} {y_{1} \left( t \right) = \mu_{1} + L_{3}^{{b_{1} }} \left( t \right) + L_{2}^{{f_{1} }} \left( t \right) + L_{3}^{{a_{1} }} \left( t \right) + e_{1} (t)} \\ {y_{2} \left( t \right) = \mu_{2} + L_{3}^{{b_{2} }} \left( t \right) + L_{1}^{{f_{2} }} \left( t \right) + L_{3}^{{a_{2} }} \left( t \right) + e_{2} (t)} \\ {y_{3} \left( t \right) = \mu_{3} + L_{3}^{{b_{3} }} \left( t \right) + L_{2}^{{f3_{{}} }} \left( t \right) + L_{2}^{{a_{3} }} \left( t \right) + e_{3} (t)} \\ \end{array} } \right.,$$

where $y_{1} \left( t \right)$, $y_{2} \left( t \right)$, and $y_{3} \left( t \right)$ are the phenotypes of BWE, BL, and BD, respectively.

#### Covariance estimates

**Fig. 1:**
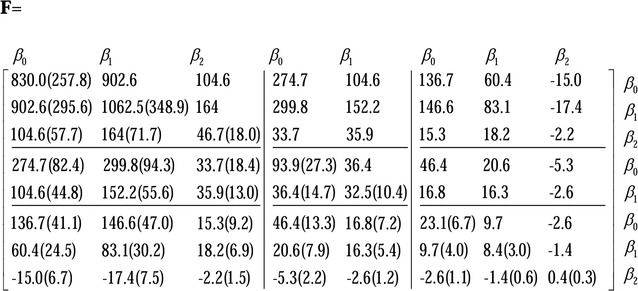
Estimates of the (co)variances (standard errors) of the random regression coefficients for family effects

**Fig. 2:**
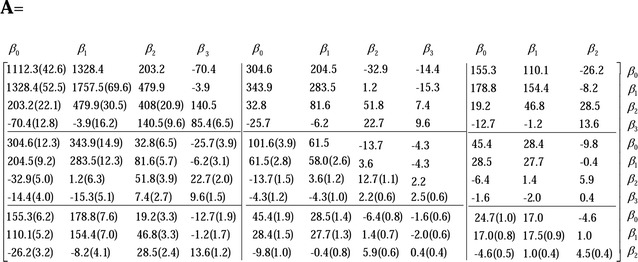
Estimates of the (co)variances (standard errors) of the random regression coefficients for additive genetic effects

Estimates of the (co)variances (standard errors) of the random regression coefficients were for the family effects and the genetic additive effects as shown in Figs. 1 and 2, respectively.

Estimates of the (co)variances (standard errors) of the random regression coefficients were for the random errors as follows:

$${\mathbf{RE}} = \left[ {\begin{array}{*{20}c} {42.06 (1.0)} & {6.2} & {5.8} \\ {6.2 (0.3)} & {7.5 (0.2)} & {1.2} \\ {5.8 (0.2)} & {1.2 (0.1)} & {3.7 (0.1)} \\ \end{array} } \right].$$

For each estimated covariance matrix of the random regression coefficients (including *β*_*0*_), the diagonal block matrices are the covariance matrices for BWE, BL, and BD, respectively, while the off-diagonal blocks are the covariance matrices between the three growth traits. All covariances of the random regression coefficients were inferred as significantly different from 0. Estimates of the genetic correlations between most of the random regression coefficients for the additive genetic effects were positive, and the few negative genetic correlations were mainly between the intercept and the cubic regression coefficients or between the linear and cubic regression coefficients. In addition, estimates of genetic covariances for the same order random regression coefficients between the pairwise growth traits were all positive and decreased with increasing order.

#### Heritability estimates

**Fig. 3:**
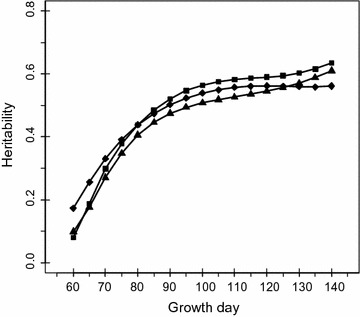
Estimated heritabilities by age for the three growth traits. Square for BWE, diamond for BL, and triangle point-up for BD

Table 3 lists the estimated ratios of family variances to phenotypic variances and heritabilities for selected days of age, using the univariate and multivariate RRM. The estimated ratios of family variances to phenotypic variances for BWE ranged from 0.259 and 0.365 and were consistently lower than these ratios for BL and BD for the period between 60 and 140 days of age. The estimated heritability obtained with the MRRM ranged from 0.080 to 0.635 for BWE, from 0.173 to 0.562 for BL, and from 0.098 to 0.610 for BD for the period between 60 and 140 days of age. Heritability estimates obtained from the MRRM were slightly lower than those obtained from univariate RRM for BWE and BL, but consistently higher for BD. Figure 3 shows that the heritabilities estimated with the MRRM increased with age for all three growth traits. Heritabilities changed most rapidly for BD and most slowly for BL. Heritabilities for BL were consistently higher than those for the other two traits from 60 to 75 days of age and those for BWE were higher than those for the other two traits from 75 days of age onward. Considering 120 days of age as the time to market, heritabilities were above moderate and similar among the three growth traits.

**Table 3:** Estimates of ratios of family to phenotypic variances (*f* ^2^) and of heritabilities (*h* ^2^) for BWE, BL and BD at selected days of age based on the univariate and multivariate RRM

Age	Univariate						Multivariate					
	BWE		BL		BD		BWE		BL		BD	
	*f* ^2^	*h* ^2^	*f* ^2^	*h* ^2^	*f* ^2^	*h* ^2^	*f* ^2^	*h* ^2^	*f* ^2^	*h* ^2^	*f* ^2^	*h* ^2^
60	0.259	0.108	0.732	0.163	0.698	0.035	0.242	0.080	0.728	0.173	0.653	0.098
65	0.334	0.169	0.654	0.256	0.628	0.141	0.356	0.187	0.656	0.256	0.611	0.177
70	0.392	0.308	0.588	0.335	0.566	0.249	0.428	0.299	0.592	0.331	0.558	0.270
75	0.421	0.401	0.538	0.396	0.527	0.331	0.456	0.378	0.542	0.391	0.514	0.348
80	0.428	0.462	0.500	0.442	0.505	0.386	0.458	0.438	0.504	0.438	0.482	0.405
85	0.422	0.507	0.472	0.478	0.492	0.422	0.447	0.485	0.476	0.474	0.460	0.446
90	0.412	0.541	0.451	0.505	0.483	0.445	0.433	0.521	0.454	0.502	0.445	0.474
95	0.403	0.565	0.435	0.527	0.476	0.461	0.421	0.547	0.438	0.523	0.436	0.494
100	0.395	0.581	0.422	0.544	0.469	0.472	0.412	0.565	0.427	0.539	0.430	0.508
105	0.392	0.590	0.413	0.556	0.463	0.480	0.406	0.576	0.420	0.550	0.426	0.518
110	0.391	0.595	0.408	0.565	0.456	0.488	0.403	0.583	0.416	0.557	0.422	0.527
115	0.392	0.597	0.406	0.570	0.449	0.496	0.402	0.587	0.415	0.561	0.418	0.535
120	0.393	0.598	0.406	0.571	0.442	0.506	0.401	0.590	0.416	0.562	0.412	0.545
125	0.393	0.600	0.409	0.570	0.433	0.520	0.398	0.595	0.418	0.561	0.404	0.556
130	0.389	0.605	0.414	0.566	0.421	0.538	0.391	0.603	0.422	0.560	0.392	0.570
135	0.380	0.615	0.420	0.563	0.404	0.560	0.379	0.616	0.424	0.559	0.377	0.588
140	0.365	0.632	0.423	0.560	0.379	0.588	0.362	0.635	0.423	0.562	0.358	0.610

#### Correlation estimates

**Fig. 4:**
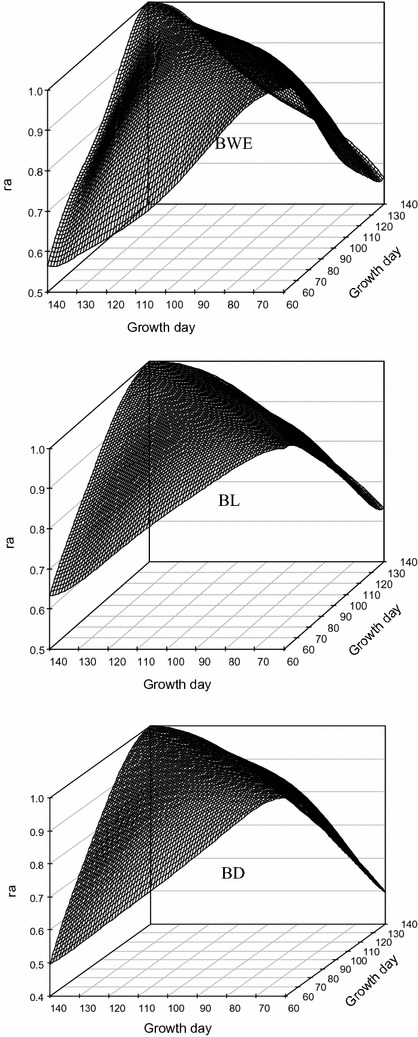
Estimates of genetic correlations between traits measured at different days of age (ra) for the three growth traits

Figure 4 presents a three-dimensional plot of estimates of genetic correlations between pairwise traits measured at different ages for each of the three growth traits from 60 to 140 days of age. Patterns of the genetic correlations over time were similar for BWE, BL, and BD. Genetic correlation estimates between measurements at adjacent days of age were close to 1 but decreased monotonously as the lag between the days of age increased. In addition, genetic correlations between measurements at early days of age were lower than those at later days of age. The lowest genetic correlations were 0.566 for BWE, 0.633 for BL, and 0.497 for BD between 60 and 140 days of age.

Table 4 includes family and phenotypic correlations between the pairwise selected growth days for BWE. Similar to the genetic correlations, phenotypic correlations approached 0.8 for the period between 75 and 120 days of age. In the earlier growth period, genetic correlations were higher than the phenotypic correlations between the pairwise days of age. The minimum family correlations were found between growth traits at 60 and 140 days of age, which were 0.573, 0.702, and 0.664 for BWE, BL, and BD, respectively. Family and phenotypic correlations for BL and BD were similar to those for BWE (see Additional file 1: Tables S1 and S2).

**Table 4:** Estimates of phenotypic (lower triangle) and family (upper triangle) correlations for body weight between pairwise measurements at selected days of age

Age	60	65	70	75	80	85	90	95	100	105	110	115	120	125	130	135	140
60	1.00	0.98	0.97	0.93	0.89	0.86	0.83	0.80	0.77	0.75	0.72	0.69	0.67	0.64	0.62	0.59	0.57
65	1.00	1.00	0.99	0.98	0.96	0.94	0.91	0.89	0.87	0.85	0.82	0.80	0.78	0.76	0.74	0.72	0.70
70	0.98	0.99	1.00	1.00	0.99	0.97	0.96	0.94	0.92	0.90	0.88	0.87	0.85	0.83	0.81	0.79	0.77
75	0.95	0.97	0.99	1.00	1.00	0.99	0.98	0.97	0.95	0.94	0.92	0.91	0.89	0.88	0.86	0.84	0.83
80	0.92	0.94	0.97	0.99	1.00	1.00	0.99	0.98	0.97	0.96	0.95	0.94	0.92	0.91	0.90	0.88	0.87
85	0.88	0.90	0.95	0.98	1.00	1.00	1.00	0.99	0.99	0.98	0.97	0.96	0.95	0.93	0.92	0.91	0.90
90	0.84	0.87	0.92	0.96	0.99	1.00	1.00	1.00	0.99	0.99	0.98	0.97	0.96	0.95	0.94	0.93	0.92
95	0.81	0.84	0.90	0.94	0.97	0.99	1.00	1.00	1.00	1.00	0.99	0.98	0.98	0.97	0.96	0.95	0.94
100	0.79	0.82	0.87	0.92	0.96	0.98	0.99	1.00	1.00	1.00	1.00	0.99	0.99	0.98	0.97	0.97	0.96
105	0.76	0.79	0.85	0.90	0.94	0.96	0.98	0.99	1.00	1.00	1.00	1.00	0.99	0.99	0.98	0.98	0.97
110	0.74	0.77	0.83	0.88	0.92	0.95	0.97	0.98	0.99	1.00	1.00	1.00	1.00	0.99	0.99	0.98	0.98
115	0.72	0.75	0.80	0.85	0.89	0.92	0.94	0.96	0.98	0.99	1.00	1.00	1.00	1.00	0.99	0.99	0.99
120	0.70	0.72	0.77	0.82	0.86	0.89	0.91	0.94	0.95	0.97	0.99	1.00	1.00	1.00	1.00	1.00	0.99
125	0.67	0.69	0.74	0.78	0.82	0.85	0.87	0.90	0.92	0.94	0.96	0.98	0.99	1.00	1.00	1.00	1.00
130	0.64	0.66	0.70	0.74	0.77	0.80	0.82	0.85	0.87	0.90	0.93	0.96	0.98	0.99	1.00	1.00	1.00
135	0.61	0.62	0.65	0.68	0.70	0.73	0.76	0.78	0.81	0.85	0.88	0.91	0.95	0.97	0.99	1.00	1.00
140	0.57	0.57	0.59	0.61	0.63	0.65	0.68	0.71	0.74	0.78	0.82	0.86	0.90	0.94	0.97	0.99	1.00

**Fig. 5:**
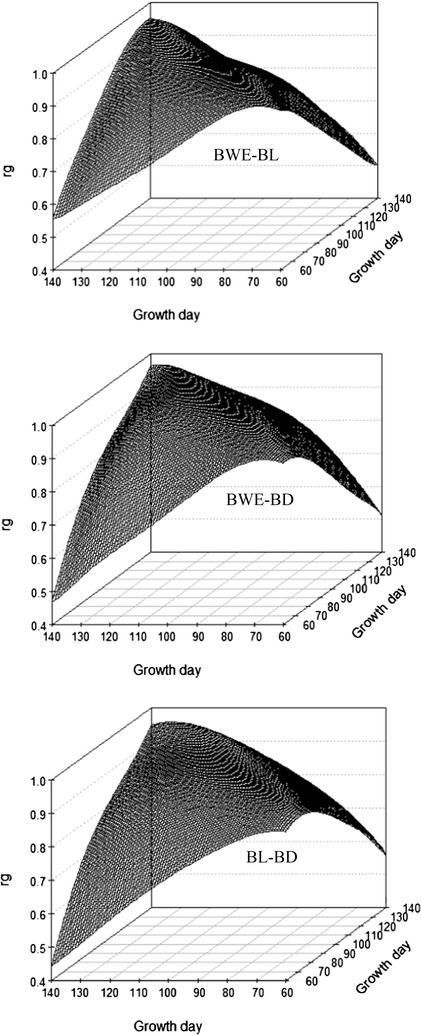
Estimates of genetic correlations (rg) between growth traits

Patterns of the genetic correlations between the pairwise growth traits were similar to those between the pairwise days of age for the same trait, but they showed lower ridges, because the genetic correlation of the same trait on the same day is 1 (Fig. 5). Genetic correlations between traits at the same age ranged from 0.89 at 140 days of age to 0.95 at 60 days of age between BWE and BL, from 0.89 to 0.96 between BWE and BD, and from 0.85 to 0.95 between BL and BD. In contrast, genetic correlations between traits at different days of age were lowest between BWE at 140 days of age and BL at 60 days of age (0.50), between BWE at 140 days of age and BD at 60 days of age (0.47), and between BL at 60 days of age and BD at 140 days of age (0.44). At harvest (120 days of age), genetic correlations were equal to 0.92 between BWE and BL, 0.94 between BWE and BD, and 0.89 between BL and BD.

**Fig. 6:**
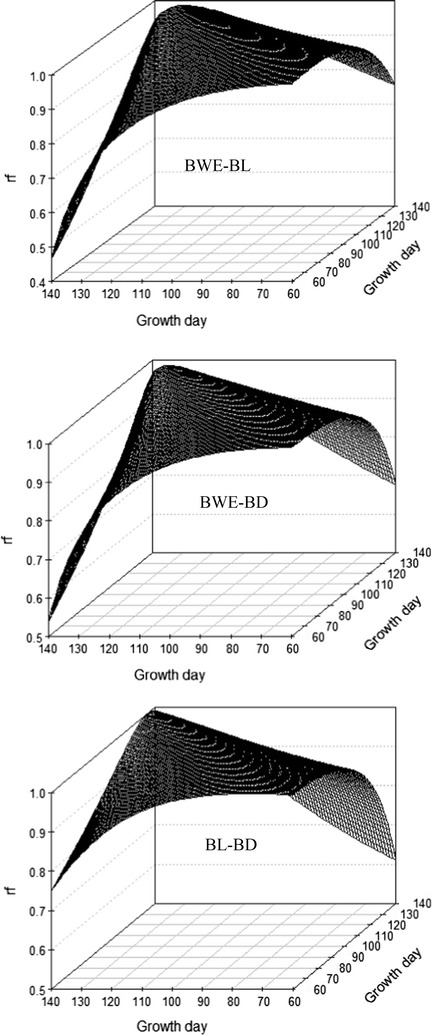
Estimates of family correlations (rf) between growth traits

Family and phenotypic correlations between the pairwise growth traits exhibited similar patterns as the genetic correlations but different magnitudes at the same compared days of age (Fig. 6). Family correlations were consistently higher than genetic correlations between BWE and BD and between BL and BD. For the same day of age, most phenotypic correlations of pairwise growth traits were lower than the corresponding genetic and family correlations (see Additional file 1: Tables S3, S4 and S5).

## Discussion

In this study, we used an MRRM to estimate heritabilities of BWE, BL, and BD based on repeated measurements during growth period, as well as genetic correlations between pairwise growth traits at specific days of age. Legendre polynomials were chosen to characterize the influence of fixed and random effects, i.e. family, additive genetic, and permanent environmental effects, on growth curves. The best MRRM was established by separately optimizing univariate RRM for each growth trait, according to the PAL. Based on the final MRRM, we found that heritabilities increased with age for all traits and that they were slightly lower than those obtained by using univariate RRM. In addition, for each trait, genetic correlations between measurements decreased monotonously with increased lag in days of age. For measurements at the same days of age, estimates of heritabilities and genetic correlations were close to those previously reported for tilapia [4–6, 8, 10–16, 18, 20–22, 33, 34].

For the selected RRM, changes in the genetic effects with day of age were optimally modeled for all analyzed traits by using Legendre polynomials of three orders. In contrast, Rutten et al. [9] and Turra et al. [27] used polynomials of two orders to fit the fixed and random effects, without selecting the orders of the polynomials for the different fixed and random effects. The non-significant variance for the permanent environmental effects showed that they have no impact on growth traits. Ratios of family variance [9] and of permanent environmental variance [27] to phenotypic variance are largely underestimated in a population with multiple families, which may be caused by the collinearity between the family and permanent environmental effects in the RRM used.

Repeated measurements during growth are required to estimate changes in both the fixed and the random effects with age when applying the RRM to genetic analysis of growth traits. More longitudinal measurements per individual would help to model growth curves with a higher goodness-of-fit and to more robustly estimate genetic parameters for random regression. However, more longitudinal measurements not only increase the experimental costs, but also affect fish growth, especially when all experimental individuals are measured together. In our experiment, the simultaneous measurement of 1600 tilapia fish incurred high labor costs and a measuring frequency of once every 15 days influenced fish growth to some extent. In future trials, the experimental population could be divided into several separately reared subpopulations, and each subpopulation could be observed in turn over more growth time-points. The extension of such measurements to more families from multiple generations would also help to identify maternal effects on early growth in tilapia within the MRRM framework.

## Conclusions

Using repeated records of growth duration from multiple families in one generation, first we introduced MRRM to genetically analyze growth curves of body weight and main morphological traits in genetically improved farmed tilapia. The optimal MRRM was chosen by comparing the univariate RRM for the analyzed traits separately via PAL. By using the covariance functions of the MRRM, changes in heritabilities with days of age and genetic correlations between lags in days of age were estimated, which could be used to carry out early selection for each trait analyzed. More importantly, for phenotypes that are measured repeatedly in aquaculture, an MRRM can enhance the efficiency of the comprehensive selection for BWE and the main morphological traits at a specific age.

## Additional file

**Additional file 1: Table S1.** Phenotypic (Lower triangle) and family (Upper triangle) correlations of body lengths between pairwise the selected days of age. **Table S2** Phenotypic (Lower triangle) and family (Upper triangle) correlations of body depths between pairwise the selected days of age. **Table S3** Phenotypic correlations between body weights and body lengths at the selected days of age. **Table S4** Phenotypic correlations between body weights and body depths at the selected days of age. **Table S5** Phenotypic correlations between body lengths and body depths at the selected days of age.
