# Supplementary material for: Multivariate random regression analysis for body weight and main morphological traits in genetically improved farmed tilapia (Oreochromis niloticus)
Source: Genet Sel Evol. 2017 Nov 2;49:80. doi: 10.1186/s12711-017-0357-7 (PMC5669032; doi:10.1186/s12711-017-0357-7)
Supplement: Supplementary file 1 — Additional file 1: Table S1. Phenotypic (Lower triangle) and family (Upper triangle) correlations of body lengths between pairwise the selected days of age. Table S2 Phenotypic (Lower triangle) and family (Upper triangle) correlations of body depths between pairwise the selected days of age. Table S3 Phenotypic correlations between body weights and body lengths at the selected days of age. Table S4 Phenotypic correlations between body weights and body depths at the selected days of age. Table S5 Phenotypic correlations between body lengths and body depths at the selected days of age. [file 12711_2017_357_MOESM1_ESM.docx]

**Table S1 Phenotypic (Lower triangle) and family (Upper triangle) correlations of body lengths between pairwise** **the selected days of age**

| Ages | 60 | 65 | 70 | 75 | 80 | 85 | 90 | 95 | 100 | 105 | 110 | 115 | 120 | 125 | 130 | 135 | 140 |
| --- | --- | --- | --- | --- | --- | --- | --- | --- | --- | --- | --- | --- | --- | --- | --- | --- | --- |
| 60 | 1.0000 | 0.9967 | 0.9874 | 0.9732 | 0.9554 | 0.9349 | 0.9128 | 0.8897 | 0.8664 | 0.8431 | 0.8204 | 0.7983 | 0.7771 | 0.7568 | 0.7375 | 0.7191 | 0.7017 |
| 65 | 0.9982 | 1.0000 | 0.9970 | 0.9887 | 0.9763 | 0.9607 | 0.9430 | 0.9240 | 0.9042 | 0.8841 | 0.8642 | 0.8447 | 0.8258 | 0.8075 | 0.7900 | 0.7733 | 0.7574 |
| 70 | 0.9933 | 0.9982 | 1.0000 | 0.9973 | 0.9901 | 0.9793 | 0.9659 | 0.9508 | 0.9345 | 0.9177 | 0.9006 | 0.8836 | 0.8670 | 0.8508 | 0.8351 | 0.8201 | 0.8056 |
| 75 | 0.9845 | 0.9921 | 0.9978 | 1.0000 | 0.9977 | 0.9915 | 0.9822 | 0.9708 | 0.9579 | 0.9441 | 0.9298 | 0.9154 | 0.9009 | 0.8868 | 0.8729 | 0.8595 | 0.8466 |
| 80 | 0.9711 | 0.9811 | 0.9906 | 0.9975 | 1.0000 | 0.9980 | 0.9927 | 0.9849 | 0.9752 | 0.9643 | 0.9527 | 0.9406 | 0.9283 | 0.9161 | 0.9040 | 0.8922 | 0.8808 |
| 85 | 0.9529 | 0.9650 | 0.9783 | 0.9898 | 0.9974 | 1.0000 | 0.9983 | 0.9938 | 0.9872 | 0.9791 | 0.9699 | 0.9601 | 0.9499 | 0.9395 | 0.9291 | 0.9189 | 0.9088 |
| 90 | 0.9308 | 0.9446 | 0.9613 | 0.9772 | 0.9897 | 0.9974 | 1.0000 | 0.9986 | 0.9948 | 0.9892 | 0.9824 | 0.9747 | 0.9664 | 0.9578 | 0.9490 | 0.9402 | 0.9315 |
| 95 | 0.9054 | 0.9207 | 0.9405 | 0.9604 | 0.9775 | 0.9901 | 0.9976 | 1.0000 | 0.9988 | 0.9956 | 0.9909 | 0.9852 | 0.9787 | 0.9717 | 0.9644 | 0.9570 | 0.9495 |
| 100 | 0.8780 | 0.8943 | 0.9168 | 0.9402 | 0.9615 | 0.9786 | 0.9907 | 0.9978 | 1.0000 | 0.9990 | 0.9963 | 0.9924 | 0.9876 | 0.9821 | 0.9762 | 0.9700 | 0.9637 |
| 105 | 0.8495 | 0.8666 | 0.8912 | 0.9177 | 0.9425 | 0.9637 | 0.9800 | 0.9914 | 0.9979 | 1.0000 | 0.9992 | 0.9969 | 0.9936 | 0.9895 | 0.9849 | 0.9799 | 0.9747 |
| 110 | 0.8207 | 0.8382 | 0.8644 | 0.8933 | 0.9213 | 0.9458 | 0.9660 | 0.9812 | 0.9918 | 0.9980 | 1.0000 | 0.9993 | 0.9974 | 0.9946 | 0.9912 | 0.9873 | 0.9830 |
| 115 | 0.7920 | 0.8094 | 0.8367 | 0.8675 | 0.8979 | 0.9253 | 0.9487 | 0.9675 | 0.9818 | 0.9918 | 0.9979 | 1.0000 | 0.9994 | 0.9978 | 0.9955 | 0.9926 | 0.9892 |
| 120 | 0.7633 | 0.7804 | 0.8082 | 0.8402 | 0.8722 | 0.9019 | 0.9278 | 0.9497 | 0.9674 | 0.9811 | 0.9912 | 0.9977 | 1.0000 | 0.9995 | 0.9982 | 0.9962 | 0.9937 |
| 125 | 0.7342 | 0.7506 | 0.7783 | 0.8106 | 0.8435 | 0.8746 | 0.9026 | 0.9270 | 0.9476 | 0.9649 | 0.9790 | 0.9899 | 0.9972 | 1.0000 | 0.9996 | 0.9984 | 0.9967 |
| 130 | 0.7038 | 0.7190 | 0.7458 | 0.7776 | 0.8105 | 0.8422 | 0.8715 | 0.8977 | 0.9209 | 0.9414 | 0.9593 | 0.9748 | 0.9875 | 0.9965 | 1.0000 | 0.9996 | 0.9987 |
| 135 | 0.6706 | 0.6842 | 0.7092 | 0.7395 | 0.7714 | 0.8027 | 0.8323 | 0.8596 | 0.8848 | 0.9080 | 0.9297 | 0.9498 | 0.9681 | 0.9839 | 0.9954 | 1.0000 | 0.9997 |
| 140 | 0.6330 | 0.6444 | 0.6666 | 0.6942 | 0.7239 | 0.7537 | 0.7825 | 0.8101 | 0.8365 | 0.8620 | 0.8870 | 0.9117 | 0.9359 | 0.9589 | 0.9791 | 0.9941 | 1.0000 |

**Table S2 Phenotypic (Lower triangle) and family (Upper triangle) correlations of body depths between pairwise the selected days of age**

| Age | 60 | 65 | 70 | 75 | 80 | 85 | 90 | 95 | 100 | 105 | 110 | 115 | 120 | 125 | 130 | 135 | 140 |
| --- | --- | --- | --- | --- | --- | --- | --- | --- | --- | --- | --- | --- | --- | --- | --- | --- | --- |
| 60 | 1.0000 | 0.9928 | 0.9753 | 0.9522 | 0.9265 | 0.9002 | 0.8743 | 0.8492 | 0.8252 | 0.8023 | 0.7805 | 0.7596 | 0.7396 | 0.7201 | 0.7012 | 0.6827 | 0.6643 |
| 65 | 0.9963 | 1.0000 | 0.9947 | 0.9819 | 0.9649 | 0.9458 | 0.9260 | 0.9062 | 0.8867 | 0.8678 | 0.8495 | 0.8317 | 0.8144 | 0.7975 | 0.7809 | 0.7645 | 0.7482 |
| 70 | 0.9870 | 0.9969 | 1.0000 | 0.9961 | 0.9867 | 0.9741 | 0.9598 | 0.9448 | 0.9294 | 0.9141 | 0.8990 | 0.8841 | 0.8694 | 0.8549 | 0.8405 | 0.8261 | 0.8117 |
| 75 | 0.9721 | 0.9875 | 0.9968 | 1.0000 | 0.9972 | 0.9902 | 0.9807 | 0.9699 | 0.9582 | 0.9461 | 0.9339 | 0.9216 | 0.9092 | 0.8969 | 0.8845 | 0.8720 | 0.8593 |
| 80 | 0.9515 | 0.9718 | 0.9871 | 0.9967 | 1.0000 | 0.9979 | 0.9926 | 0.9854 | 0.9770 | 0.9678 | 0.9581 | 0.9481 | 0.9378 | 0.9274 | 0.9168 | 0.9060 | 0.8949 |
| 85 | 0.9256 | 0.9504 | 0.9713 | 0.9871 | 0.9968 | 1.0000 | 0.9984 | 0.9944 | 0.9887 | 0.9820 | 0.9746 | 0.9666 | 0.9583 | 0.9496 | 0.9406 | 0.9313 | 0.9217 |
| 90 | 0.8953 | 0.9240 | 0.9501 | 0.9717 | 0.9875 | 0.9969 | 1.0000 | 0.9988 | 0.9956 | 0.9911 | 0.9857 | 0.9795 | 0.9728 | 0.9657 | 0.9582 | 0.9502 | 0.9419 |
| 95 | 0.8619 | 0.8939 | 0.9246 | 0.9515 | 0.9730 | 0.9882 | 0.9971 | 1.0000 | 0.9990 | 0.9965 | 0.9928 | 0.9883 | 0.9831 | 0.9773 | 0.9711 | 0.9644 | 0.9572 |
| 100 | 0.8264 | 0.8611 | 0.8958 | 0.9274 | 0.9540 | 0.9746 | 0.9890 | 0.9973 | 1.0000 | 0.9992 | 0.9971 | 0.9941 | 0.9902 | 0.9857 | 0.9806 | 0.9751 | 0.9689 |
| 105 | 0.7898 | 0.8266 | 0.8645 | 0.9001 | 0.9311 | 0.9566 | 0.9760 | 0.9895 | 0.9974 | 1.0000 | 0.9993 | 0.9976 | 0.9950 | 0.9916 | 0.9876 | 0.9831 | 0.9779 |
| 110 | 0.7527 | 0.7909 | 0.8313 | 0.8701 | 0.9048 | 0.9344 | 0.9584 | 0.9767 | 0.9896 | 0.9973 | 1.0000 | 0.9994 | 0.9979 | 0.9956 | 0.9926 | 0.9890 | 0.9848 |
| 115 | 0.7152 | 0.7542 | 0.7962 | 0.8373 | 0.8749 | 0.9080 | 0.9358 | 0.9584 | 0.9761 | 0.9889 | 0.9971 | 1.0000 | 0.9995 | 0.9982 | 0.9961 | 0.9934 | 0.9900 |
| 120 | 0.6771 | 0.7160 | 0.7587 | 0.8010 | 0.8406 | 0.8763 | 0.9073 | 0.9337 | 0.9557 | 0.9736 | 0.9873 | 0.9965 | 1.0000 | 0.9996 | 0.9984 | 0.9965 | 0.9939 |
| 125 | 0.6375 | 0.6755 | 0.7176 | 0.7600 | 0.8005 | 0.8376 | 0.8710 | 0.9006 | 0.9265 | 0.9492 | 0.9686 | 0.9844 | 0.9956 | 1.0000 | 0.9996 | 0.9985 | 0.9967 |
| 130 | 0.5951 | 0.6310 | 0.6713 | 0.7124 | 0.7523 | 0.7898 | 0.8245 | 0.8563 | 0.8856 | 0.9127 | 0.9378 | 0.9605 | 0.9800 | 0.9943 | 1.0000 | 0.9996 | 0.9986 |
| 135 | 0.5485 | 0.5810 | 0.6179 | 0.6561 | 0.6938 | 0.7301 | 0.7648 | 0.7979 | 0.8297 | 0.8607 | 0.8911 | 0.9209 | 0.9491 | 0.9741 | 0.9926 | 1.0000 | 0.9997 |
| 140 | 0.4965 | 0.5243 | 0.5561 | 0.5895 | 0.6233 | 0.6568 | 0.6899 | 0.7228 | 0.7561 | 0.7901 | 0.8253 | 0.8617 | 0.8988 | 0.9349 | 0.9672 | 0.9908 | 1.0000 |

**Table S3 Phenotypic correlations between body weights and body lengths at** **the selected days of age**

| Age | 60 | 65 | 70 | 75 | 80 | 85 | 90 | 95 | 100 | 105 | 110 | 115 | 120 | 125 | 130 | 135 | 140 |
| --- | --- | --- | --- | --- | --- | --- | --- | --- | --- | --- | --- | --- | --- | --- | --- | --- | --- |
| 60 | 0.7786 | 0.7118 | 0.6997 | 0.6835 | 0.6645 | 0.6434 | 0.6211 | 0.5981 | 0.5748 | 0.5517 | 0.5290 | 0.5066 | 0.4846 | 0.4628 | 0.4407 | 0.4180 | 0.3942 |
| 65 | 0.8045 | 0.8531 | 0.8037 | 0.7938 | 0.7797 | 0.7625 | 0.7430 | 0.7219 | 0.6999 | 0.6774 | 0.6547 | 0.6319 | 0.6089 | 0.5855 | 0.5613 | 0.5357 | 0.5080 |
| 70 | 0.8406 | 0.8528 | 0.8901 | 0.8536 | 0.8458 | 0.8341 | 0.8193 | 0.8021 | 0.7832 | 0.7630 | 0.7420 | 0.7201 | 0.6974 | 0.6734 | 0.6478 | 0.6199 | 0.5888 |
| 75 | 0.8483 | 0.8677 | 0.8783 | 0.9080 | 0.8808 | 0.8748 | 0.8651 | 0.8523 | 0.8372 | 0.8202 | 0.8015 | 0.7814 | 0.7597 | 0.7361 | 0.7100 | 0.6807 | 0.6472 |
| 80 | 0.8406 | 0.8658 | 0.8825 | 0.8924 | 0.9166 | 0.8960 | 0.8911 | 0.8828 | 0.8715 | 0.8578 | 0.8420 | 0.8240 | 0.8040 | 0.7813 | 0.7555 | 0.7257 | 0.6910 |
| 85 | 0.8256 | 0.8554 | 0.8771 | 0.8920 | 0.9012 | 0.9211 | 0.9049 | 0.9007 | 0.8931 | 0.8827 | 0.8697 | 0.8543 | 0.8362 | 0.8150 | 0.7902 | 0.7608 | 0.7259 |
| 90 | 0.8075 | 0.8411 | 0.8668 | 0.8860 | 0.8994 | 0.9076 | 0.9239 | 0.9107 | 0.9066 | 0.8994 | 0.8894 | 0.8765 | 0.8607 | 0.8415 | 0.8182 | 0.7899 | 0.7556 |
| 95 | 0.7888 | 0.8253 | 0.8543 | 0.8769 | 0.8940 | 0.9058 | 0.9129 | 0.9262 | 0.9150 | 0.9108 | 0.9036 | 0.8934 | 0.8801 | 0.8632 | 0.8420 | 0.8154 | 0.7825 |
| 100 | 0.7703 | 0.8090 | 0.8407 | 0.8662 | 0.8863 | 0.9012 | 0.9114 | 0.9174 | 0.9282 | 0.9184 | 0.9140 | 0.9067 | 0.8960 | 0.8817 | 0.8630 | 0.8388 | 0.8079 |
| 105 | 0.7520 | 0.7925 | 0.8262 | 0.8541 | 0.8767 | 0.8942 | 0.9072 | 0.9159 | 0.9210 | 0.9300 | 0.9212 | 0.9167 | 0.9090 | 0.8976 | 0.8817 | 0.8604 | 0.8323 |
| 110 | 0.7336 | 0.7752 | 0.8105 | 0.8403 | 0.8649 | 0.8847 | 0.9001 | 0.9114 | 0.9190 | 0.9235 | 0.9312 | 0.9234 | 0.9188 | 0.9106 | 0.8981 | 0.8802 | 0.8555 |
| 115 | 0.7142 | 0.7566 | 0.7930 | 0.8241 | 0.8503 | 0.8720 | 0.8894 | 0.9030 | 0.9132 | 0.9203 | 0.9246 | 0.9316 | 0.9249 | 0.9202 | 0.9115 | 0.8974 | 0.8768 |
| 120 | 0.6931 | 0.7357 | 0.7726 | 0.8045 | 0.8319 | 0.8550 | 0.8742 | 0.8899 | 0.9023 | 0.9121 | 0.9193 | 0.9240 | 0.9309 | 0.9254 | 0.9207 | 0.9111 | 0.8952 |
| 125 | 0.6693 | 0.7115 | 0.7484 | 0.7807 | 0.8088 | 0.8329 | 0.8535 | 0.8710 | 0.8856 | 0.8978 | 0.9078 | 0.9158 | 0.9216 | 0.9289 | 0.9246 | 0.9199 | 0.9091 |
| 130 | 0.6421 | 0.6834 | 0.7197 | 0.7517 | 0.7800 | 0.8048 | 0.8265 | 0.8454 | 0.8619 | 0.8764 | 0.8893 | 0.9005 | 0.9100 | 0.9174 | 0.9254 | 0.9223 | 0.9172 |
| 135 | 0.6110 | 0.6508 | 0.6859 | 0.7173 | 0.7453 | 0.7703 | 0.7926 | 0.8126 | 0.8307 | 0.8474 | 0.8628 | 0.8772 | 0.8904 | 0.9020 | 0.9113 | 0.9203 | 0.9182 |
| 140 | 0.5761 | 0.6137 | 0.6472 | 0.6773 | 0.7046 | 0.7293 | 0.7518 | 0.7726 | 0.7921 | 0.8106 | 0.8285 | 0.8458 | 0.8626 | 0.8784 | 0.8926 | 0.9040 | 0.9138 |

**Table S4 Phenotypic correlations between body weights and body depths at the selected days of age**

| Age | 60 | 65 | 70 | 75 | 80 | 85 | 90 | 95 | 100 | 105 | 110 | 115 | 120 | 125 | 130 | 135 | 140 |
| --- | --- | --- | --- | --- | --- | --- | --- | --- | --- | --- | --- | --- | --- | --- | --- | --- | --- |
| 60 | 0.8101 | 0.6872 | 0.6765 | 0.6611 | 0.6442 | 0.6269 | 0.6097 | 0.5924 | 0.5747 | 0.5564 | 0.5371 | 0.5163 | 0.4938 | 0.4693 | 0.4427 | 0.4138 | 0.3828 |
| 65 | 0.7627 | 0.8686 | 0.7806 | 0.7734 | 0.7622 | 0.7490 | 0.7346 | 0.7193 | 0.7027 | 0.6848 | 0.6651 | 0.6433 | 0.6190 | 0.5919 | 0.5619 | 0.5287 | 0.4926 |
| 70 | 0.7915 | 0.8243 | 0.9013 | 0.8379 | 0.8331 | 0.8249 | 0.8143 | 0.8018 | 0.7874 | 0.7710 | 0.7521 | 0.7306 | 0.7060 | 0.6780 | 0.6464 | 0.6110 | 0.5720 |
| 75 | 0.7941 | 0.8403 | 0.8628 | 0.9200 | 0.8733 | 0.8699 | 0.8632 | 0.8539 | 0.8421 | 0.8277 | 0.8105 | 0.7901 | 0.7663 | 0.7385 | 0.7067 | 0.6707 | 0.6305 |
| 80 | 0.7830 | 0.8399 | 0.8709 | 0.8868 | 0.9303 | 0.8947 | 0.8918 | 0.8857 | 0.8766 | 0.8647 | 0.8495 | 0.8309 | 0.8084 | 0.7818 | 0.7508 | 0.7152 | 0.6752 |
| 85 | 0.7653 | 0.8306 | 0.8687 | 0.8903 | 0.9018 | 0.9357 | 0.9075 | 0.9045 | 0.8983 | 0.8889 | 0.8760 | 0.8595 | 0.8390 | 0.8141 | 0.7845 | 0.7502 | 0.7112 |
| 90 | 0.7451 | 0.8171 | 0.8607 | 0.8871 | 0.9027 | 0.9115 | 0.9384 | 0.9153 | 0.9118 | 0.9050 | 0.8947 | 0.8806 | 0.8623 | 0.8396 | 0.8120 | 0.7796 | 0.7422 |
| 95 | 0.7246 | 0.8015 | 0.8497 | 0.8800 | 0.8992 | 0.9112 | 0.9180 | 0.9398 | 0.9202 | 0.9160 | 0.9084 | 0.8968 | 0.8811 | 0.8609 | 0.8358 | 0.8056 | 0.7704 |
| 100 | 0.7044 | 0.7850 | 0.8367 | 0.8703 | 0.8926 | 0.9075 | 0.9172 | 0.9228 | 0.9406 | 0.9235 | 0.9186 | 0.9098 | 0.8969 | 0.8795 | 0.8572 | 0.8298 | 0.7972 |
| 105 | 0.6845 | 0.7676 | 0.8220 | 0.8583 | 0.8833 | 0.9009 | 0.9133 | 0.9216 | 0.9264 | 0.9412 | 0.9258 | 0.9201 | 0.9103 | 0.8959 | 0.8767 | 0.8524 | 0.8228 |
| 110 | 0.6647 | 0.7491 | 0.8054 | 0.8439 | 0.8712 | 0.8912 | 0.9061 | 0.9171 | 0.9247 | 0.9290 | 0.9416 | 0.9275 | 0.9209 | 0.9100 | 0.8942 | 0.8733 | 0.8472 |
| 115 | 0.6440 | 0.7288 | 0.7862 | 0.8264 | 0.8556 | 0.8778 | 0.8951 | 0.9087 | 0.9190 | 0.9264 | 0.9306 | 0.9413 | 0.9283 | 0.9210 | 0.9090 | 0.8920 | 0.8697 |
| 120 | 0.6219 | 0.7059 | 0.7636 | 0.8048 | 0.8356 | 0.8598 | 0.8793 | 0.8954 | 0.9085 | 0.9189 | 0.9264 | 0.9307 | 0.9401 | 0.9280 | 0.9201 | 0.9072 | 0.8892 |
| 125 | 0.5974 | 0.6794 | 0.7367 | 0.7784 | 0.8103 | 0.8361 | 0.8577 | 0.8762 | 0.8921 | 0.9055 | 0.9163 | 0.9243 | 0.9288 | 0.9372 | 0.9260 | 0.9176 | 0.9042 |
| 130 | 0.5699 | 0.6488 | 0.7047 | 0.7463 | 0.7790 | 0.8061 | 0.8296 | 0.8503 | 0.8688 | 0.8852 | 0.8994 | 0.9109 | 0.9195 | 0.9246 | 0.9322 | 0.9219 | 0.9133 |
| 135 | 0.5391 | 0.6137 | 0.6674 | 0.7083 | 0.7412 | 0.7693 | 0.7943 | 0.8171 | 0.8380 | 0.8573 | 0.8747 | 0.8899 | 0.9025 | 0.9119 | 0.9175 | 0.9246 | 0.9152 |
| 140 | 0.5051 | 0.5743 | 0.6251 | 0.6645 | 0.6972 | 0.7259 | 0.7521 | 0.7766 | 0.7998 | 0.8217 | 0.8422 | 0.8609 | 0.8773 | 0.8910 | 0.9012 | 0.9074 | 0.9141 |

**Table S5 Phenotypic correlations between body lengths and body depths at** **the selected days of age**

| Age | 60 | 65 | 70 | 75 | 80 | 85 | 90 | 95 | 100 | 105 | 110 | 115 | 120 | 125 | 130 | 135 | 140 |
| --- | --- | --- | --- | --- | --- | --- | --- | --- | --- | --- | --- | --- | --- | --- | --- | --- | --- |
| 60 | 0.8708 | 0.8551 | 0.8521 | 0.8414 | 0.8274 | 0.8119 | 0.7956 | 0.7787 | 0.7609 | 0.7419 | 0.7213 | 0.6986 | 0.6734 | 0.6455 | 0.6144 | 0.5802 | 0.5428 |
| 65 | 0.8355 | 0.8863 | 0.8682 | 0.8650 | 0.8566 | 0.8455 | 0.8328 | 0.8185 | 0.8029 | 0.7854 | 0.7659 | 0.7438 | 0.7189 | 0.6907 | 0.6590 | 0.6237 | 0.5849 |
| 70 | 0.8227 | 0.8599 | 0.8964 | 0.8790 | 0.8761 | 0.8694 | 0.8601 | 0.8488 | 0.8355 | 0.8201 | 0.8021 | 0.7813 | 0.7573 | 0.7296 | 0.6981 | 0.6627 | 0.6233 |
| 75 | 0.8055 | 0.8525 | 0.8759 | 0.9038 | 0.8879 | 0.8854 | 0.8797 | 0.8714 | 0.8607 | 0.8475 | 0.8315 | 0.8123 | 0.7897 | 0.7631 | 0.7325 | 0.6976 | 0.6585 |
| 80 | 0.7852 | 0.8407 | 0.8710 | 0.8866 | 0.9092 | 0.8950 | 0.8927 | 0.8874 | 0.8794 | 0.8686 | 0.8548 | 0.8377 | 0.8168 | 0.7918 | 0.7626 | 0.7288 | 0.6906 |
| 85 | 0.7628 | 0.8256 | 0.8621 | 0.8828 | 0.8940 | 0.9130 | 0.9003 | 0.8980 | 0.8927 | 0.8844 | 0.8729 | 0.8580 | 0.8392 | 0.8162 | 0.7886 | 0.7564 | 0.7196 |
| 90 | 0.7391 | 0.8080 | 0.8498 | 0.8751 | 0.8903 | 0.8990 | 0.9153 | 0.9038 | 0.9012 | 0.8955 | 0.8865 | 0.8740 | 0.8574 | 0.8366 | 0.8111 | 0.7808 | 0.7458 |
| 95 | 0.7147 | 0.7887 | 0.8351 | 0.8644 | 0.8832 | 0.8951 | 0.9023 | 0.9165 | 0.9058 | 0.9027 | 0.8962 | 0.8861 | 0.8720 | 0.8536 | 0.8304 | 0.8024 | 0.7694 |
| 100 | 0.6902 | 0.7683 | 0.8185 | 0.8514 | 0.8733 | 0.8883 | 0.8982 | 0.9043 | 0.9169 | 0.9065 | 0.9027 | 0.8951 | 0.8836 | 0.8677 | 0.8470 | 0.8214 | 0.7907 |
| 105 | 0.6658 | 0.7472 | 0.8007 | 0.8366 | 0.8614 | 0.8791 | 0.8917 | 0.9003 | 0.9056 | 0.9166 | 0.9064 | 0.9014 | 0.8925 | 0.8792 | 0.8612 | 0.8382 | 0.8100 |
| 110 | 0.6420 | 0.7259 | 0.7819 | 0.8204 | 0.8479 | 0.8680 | 0.8830 | 0.8941 | 0.9019 | 0.9064 | 0.9159 | 0.9054 | 0.8992 | 0.8886 | 0.8733 | 0.8531 | 0.8276 |
| 115 | 0.6187 | 0.7043 | 0.7624 | 0.8031 | 0.8327 | 0.8551 | 0.8725 | 0.8859 | 0.8960 | 0.9031 | 0.9069 | 0.9148 | 0.9037 | 0.8960 | 0.8836 | 0.8662 | 0.8436 |
| 120 | 0.5958 | 0.6824 | 0.7420 | 0.7845 | 0.8161 | 0.8405 | 0.8599 | 0.8756 | 0.8880 | 0.8974 | 0.9038 | 0.9068 | 0.9131 | 0.9012 | 0.8917 | 0.8773 | 0.8577 |
| 125 | 0.5732 | 0.6600 | 0.7205 | 0.7643 | 0.7975 | 0.8237 | 0.8450 | 0.8628 | 0.8774 | 0.8892 | 0.8981 | 0.9038 | 0.9059 | 0.9104 | 0.8975 | 0.8862 | 0.8697 |
| 130 | 0.5503 | 0.6365 | 0.6974 | 0.7421 | 0.7765 | 0.8042 | 0.8273 | 0.8469 | 0.8637 | 0.8778 | 0.8891 | 0.8974 | 0.9023 | 0.9034 | 0.9061 | 0.8919 | 0.8787 |
| 135 | 0.5266 | 0.6113 | 0.6718 | 0.7169 | 0.7521 | 0.7811 | 0.8056 | 0.8270 | 0.8458 | 0.8621 | 0.8758 | 0.8867 | 0.8944 | 0.8985 | 0.8983 | 0.8991 | 0.8837 |
| 140 | 0.5015 | 0.5836 | 0.6430 | 0.6879 | 0.7235 | 0.7533 | 0.7791 | 0.8020 | 0.8225 | 0.8408 | 0.8569 | 0.8703 | 0.8808 | 0.8879 | 0.8909 | 0.8895 | 0.8884 |
